# Removing barriers to utilisation of support services for abused female adolescents in Nigeria slums

**DOI:** 10.1093/eurpub/ckac131.455

**Published:** 2022-10-25

**Authors:** OO Ikuteyijo, AI Akinyemi, S Merten, MD Fetters

**Affiliations:** 1 Society, Gender and Health, Department of Epidemiology and Public Health, Swiss Tropical and Public Health Institute, University of Basel Switzerland, Basel, Switzerland; 2 Center for African Studies, University of Basel, Basel, Switzerland; 3 Mixed Method Program and Faculty of Medicine, University of Michigan, Michigan, USA; 4 Department of Demography and Social Statistic, Obafemi Awowolo University, Ile Ife, Nigeria

## Abstract

**Background:**

Female adolescents in urban slums experience a plethora of violence. The inherent health inequalities in the urban slums also present barriers to adolescents’ access to support services that can alleviate the impact of violence and bring perpetrators to justice. Health facilities can play key proactive roles in facilitating effective responses to address the problems of violence. This research sought to answer the questions: what support services are available to female adolescents in the event of violence; what are the barriers to accessing these services; and what roles can health workers play in removing these barriers?

**Methods:**

The study used an ethnographic approach involving 40 in-depth interviews and 9 focus group discussions with female adolescents, 17 in-depth interviews were conducted with health providers and community leaders. The study setting comprised intentionally sampled slum communities in Lagos and Oyo states, southwest Nigeria. Thematic data analysis was conducted to address the study questions.

**Results:**

Potential support services available to female adolescents in the study setting included the Community Development Association, police, family members, and health facilities. Identified barriers to utilizing available support services included stigmatization, non-formalization of police reports of violence, ambiguous attitudes of health workers to abused adolescents, and unfamiliarity on the part of adolescents. Although resources are available to adolescents in the event of violence, the lack of coordination of services has led to gross inefficiency for intervening.

**Conclusions:**

To address the inefficiency of services, the health sector is best positioned to ensure synergy among the key stakeholders to reduce stigma and stop abuse experience among adolescent girls. Beyond a reactionary, curative approach, health providers need to play a preventive role through education, advocacy, and coordination of interventions at the community level.

**Key messages:**

• Health workers at primary health facilities need to support adolescents who experience violence, especially those who using their services.

• In addition, health workers are best positioned to create synergy among available support services to alleviate and mitigate the impact of violence on female adolescents in the community.

